# Shot-by-shot characterization of focused X-ray free electron laser pulses

**DOI:** 10.1038/s41598-018-19179-3

**Published:** 2018-01-16

**Authors:** Amane Kobayashi, Yuki Sekiguchi, Tomotaka Oroguchi, Masaki Yamamoto, Masayoshi Nakasako

**Affiliations:** 1 Department of Physics, Faculty of Science and Technology, Keio University, 3-14-1 Hiyoshi, Kohoku-ku, Yokohama, Kanagawa 223-8522 Japan; 2 RIKEN SPring-8 Center, 1-1-1 Kouto, Sayo, Hyogo 679-5148 Japan

## Abstract

X-ray free electron lasers (XFEL) provide intense and almost coherent X-ray pulses. They are used for various experiments investigating physical and chemical properties in materials and biological science because of their complete coherence, high intensity, and very short pulse width. In XFEL experiments, specimens are irradiated by XFEL pulses focused by mirror optics. The focused pulse is too intense to measure its coherence by placing an X-ray detector on the focal spot. Previously, a method was proposed for evaluating the coherence of focused pulses from the visibility of the diffraction intensity of colloidal particles by the speckle visibility spectroscopy (SVS). However, the visibility cannot be determined exactly because the diffraction intensity is integrated into each finite size detector pixel. Here, we propose a method to evaluate the coherence of each XFEL pulse by using SVS in combination with a theory for exact sampling of the diffraction pattern and a technique of multiplying the diffraction data by a Gaussian masks, which reduces the influence of data missing in small-angle regions due to the presence of a direct beamstop. We also introduce a method for characterizing the shot-by-shot size of each XFEL pulse by analysing the X-ray irradiated area.

## Introduction

An X-ray free electron laser (XFEL) is a light source providing X-rays with complete spatial coherence, high intensity, and very short pulse duration. By utilizing this light source, new physical and chemical properties of materials have been revealed in recent years. For instance, XFEL pulses with high intensity and very short duration were utilized in fast pump-probe experiments^1–3^, crystal structure analysis of large membrane proteins without radiation damage^4–6^, structural investigations of ultrafast dynamics of atoms in a molecule^7^, and coherent X-ray diffraction imaging (CXDI) of noncrystalline particles with small scattering cross sections^8–17^.

**Figure 1:**
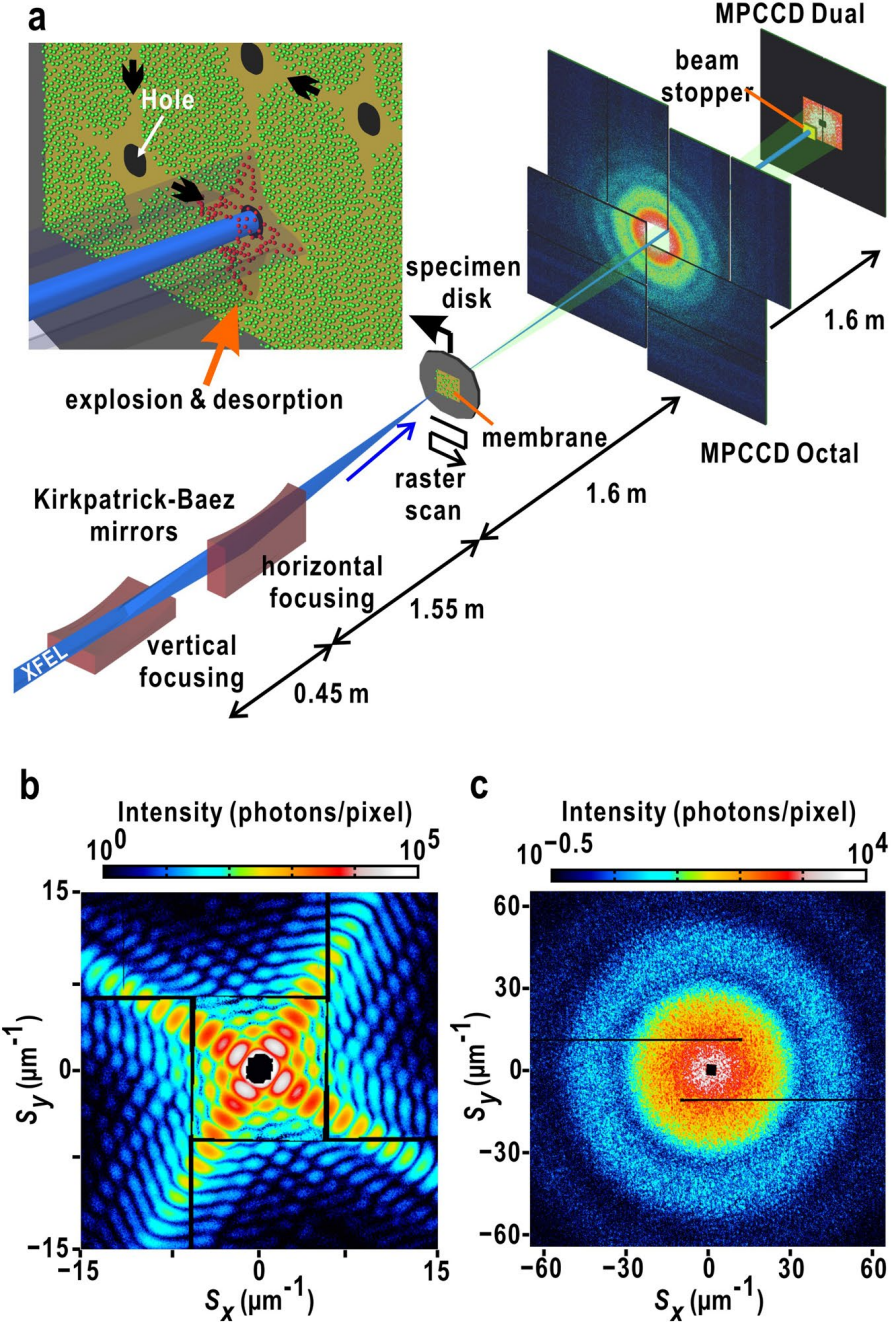
(**a**) Schematic illustration of the experimental setup for SVS measurement of XFEL pulses focused by K-B mirrors^34^. A specimen disk carrying uniformly and densely dispersed gold colloid particles is scanned against XFEL pulse trains, and then the diffraction patterns are recorded by using two MPCCD detectors^35^. The upper left illustration is a magnified view of the membrane. Green dots indicate absorbed specimen particles and red dots indicate desorbed particles due to X-ray irradiation. Diffraction patterns from a cuboid-shaped cuprous oxide particle are used to confirm the visibility (**b**), and from densely dispersed gold colloidal particles for the SVS measurement (**c**). In panels (b) and (c), diffraction intensities are plotted as the logarithmic scale as indicated by the scale bars at the top of the diffraction patterns.

Focusing-mirror optics, such as Kirkpatrick-Baez (K-B) mirrors^18^, are used in CXDI experiments requiring strong XFEL pulses with high spatial coherence (Fig. 1(a)). The average intensity and beam profile can be measured for hundreds of heavily attenuated XFEL pulses with a knife-edge scan^14,15^. The high spatial coherence of the focused XFEL pulse can be observed, for instance, by the diffraction pattern with good visibility from a single cuboid-shaped cuprous oxide particle^14,17^ (Fig. 1(b)). Although shot-by-shot characterization of the focused X-ray pulse is necessary with respect to spatial coherence and size, focused XFEL pulses with intensities exceeding 10^10^ X-ray photons vaporize specimen particles through Coulombic explosion immediately after the particles diffract the X-rays. Therefore, observation of the diffraction patterns from the same particle is impossible.

Recently, the use of speckle visibility spectroscopy (SVS)^19–21^ has been proposed to monitor the spatial coherence of focused XFEL pulses from diffraction patterns collected from gold colloidal particles filled in capillary tubes^20,21^. We applied the proposed SVS analysis to diffraction patterns collected by scanning a silicon nitride membrane with densely dispersed gold colloidal particles at SACLA (Fig. 1(c)). After the SVS measurement, the diffraction patterns of cuprous oxide particles were collected (Fig. 1(b)), because the diffraction patterns comprising cross-shaped flares with a train of the large speckle peaks were suitable for easily evaluating the visibility. The evaluated visibility from the patterns (Supplementary Information S1) was inconsistent with that estimated by the SVS measurement.

In this study, to understand the discrepancy described above, we propose a procedure to estimate the spatial coherence of each focused XFEL pulse from the diffraction patterns of gold colloidal particles by combining the exact sampling method for the diffraction pattern^22^ and a mask technique developed in the dark-field phase-retrieval method^23^. Then, the beam size contributing to the diffraction patterns is estimated from the autocorrelation function and compared to the area damaged by the focused XFEL pulses. Here, we report the details of the method, including the experimental procedures, and evaluate the quality of the focused XFEL pulses provided at SACLA.

## Theoretical background

### Visibility and speckle contrast

In SVS, visibility of diffraction patterns is evaluated from the speckle contrast, *C*, defined as

1 $$C=\frac{{\langle {I}^{2}\rangle }_{T}-{{\langle I\rangle }_{T}}^{2}}{{{\langle I\rangle }_{T}}^{2}},\quad {\langle I\rangle }_{T}=\frac{1}{T}{\int }_{0}^{T}{I}_{i}(t)\,dt,$$

where *I*_*i*_(t) is diffraction intensity of particles at time *t* in *i*-th pixel. 〈*I*〉_*T*_ is the average of diffraction intensity in an period of time *T*. Under an ideally coherent illumination, speckle contrast is equal to 1. If we observe diffraction intensities *I*_*i*_(*t*_1_) = *I*_min_ and *I*_*i*_(*t*_2_) = *I*_max_. The intensity 〈*I*〉_*T*_ and 〈*I*^2^〉_*T*_ is expressed as,

2 $${\langle I\rangle }_{T}=\frac{{I}_{\min }+{I}_{\max }}{2},\,{\langle {I}^{2}\rangle }_{T}=\frac{{{I}_{\min }}^{2}+{{I}_{\max }}^{2}}{2}.$$

Then, the speckle contrast is rewritten as

3 $$C={(\frac{{I}_{\min }-{I}_{\max }}{{I}_{\min }+{I}_{\max }})}^{2}$$

Therefore, the speckle contrast is defined as the square of visibility, and also as the square of the normalized variance of intensity fluctuation.

For focused XFEL pulses, it is almost impossible to measure the variation of diffraction intensity to calculate the average 〈*I*〉_*T*_, because specimen particles are destroyed by single X-ray pulses immediately after emitting diffraction waves. Thus, the speckle contrast is evaluated by measuring the spatial variation of diffraction intensities within a single-shot diffraction pattern from nanometer-sized colloidal particles instead the time-dependent variation^20,21^.

### Speckle contrast measurement for a number of spherical particles

**Figure 2:**
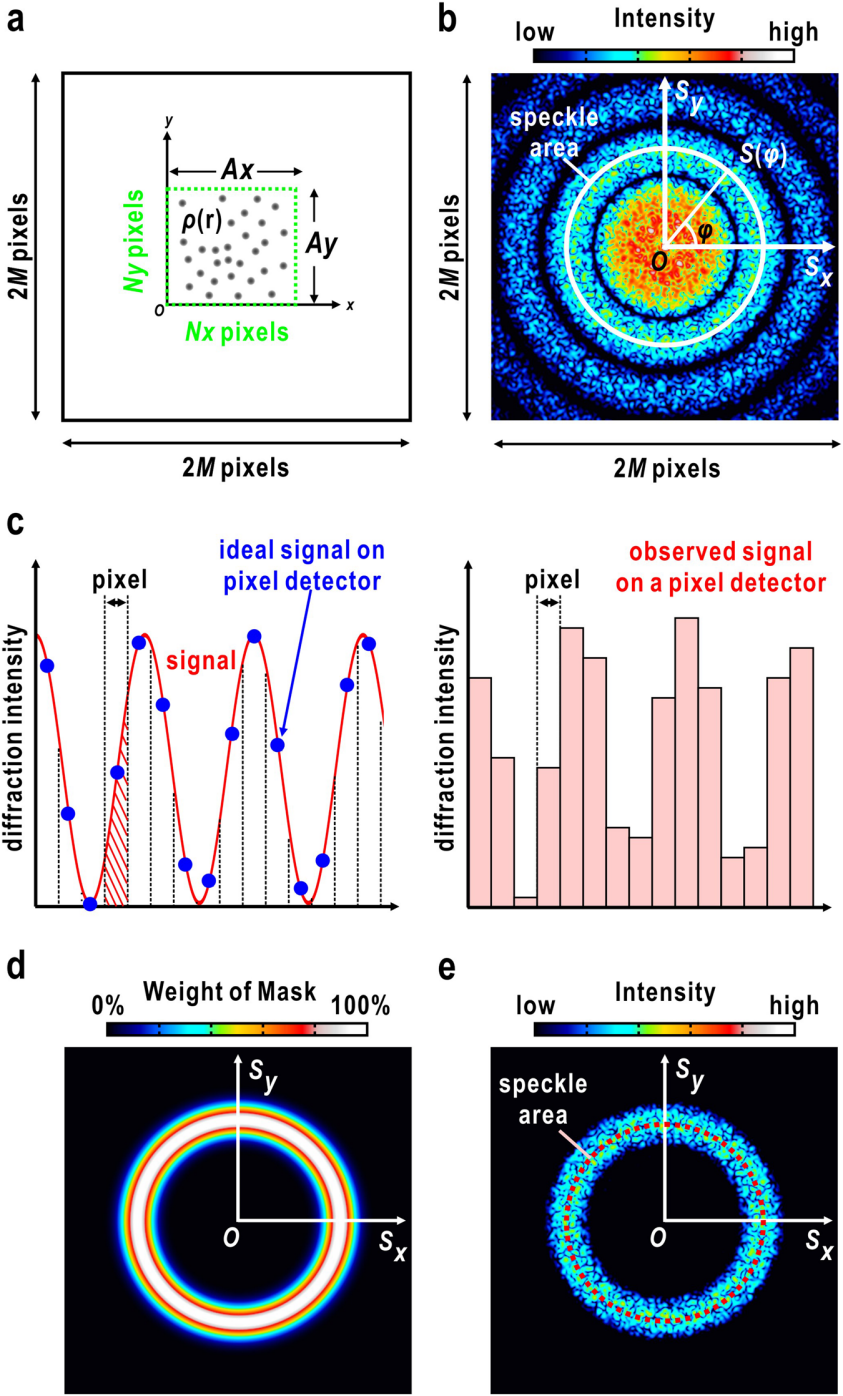
(**a**) A specimen composed of many colloidal particles, and (**b**) the simulated diffraction pattern (right panel). Theoretical details are described in the main text. (**c**) Sampling of diffraction patterns by pixels in an area detector. Diffraction intensities from the ideal case (left panel) and collected by an area detector (right panel) are compared. (**d**) Annular-shaped mask used to significantly reduce the aliasing in the Fourier transform of the diffraction pattern. (**e)** A simulated diffraction pattern of colloidal particles, to which an annular-shaped mask is applied.

When a number of spherical particles with radius *D* and average electron density *ρ*_0_ are irradiated by a focused XFEL pulse with a wavelength of *λ* (Fig. 2(a)), the diffraction intensity at scattering vector ***S*** with scattering angle 2*θ* (│***S***│ = 2sin*θ*/*λ*) is expressed as

4 $$I({\boldsymbol{S}})\,\propto {|\sum _{n}\exp (2\pi i{{\boldsymbol{L}}}_{n}\cdot {\boldsymbol{S}})|}^{2}\cdot {|\frac{4\pi }{{\rm{3}}}{D}^{3}{\rho }_{0}\frac{{J}_{1}(2\pi SD)}{2\pi SD}|}^{2},$$

where ***L***_*n*_ is the position of the *n*^th^ particle in the irradiation area. *J*_1_(*x*) is the first-order cylindrical Bessel function. A diffraction pattern is composed of the concentric fringe rings from the structure factor of a single particle and many speckle peaks caused by the interference between diffracted waves from particles (Fig. 2(b)).

The speckle contrast of the diffraction pattern is evaluated as

5 $$C=\frac{{\langle {I}^{2}\rangle }_{{\rm{Area}}}-{{\langle I\rangle }_{{\rm{Area}}}}^{2}}{{{\langle I\rangle }_{{\rm{Area}}}}^{2}}$$

The area average 〈〉_Area_ is taken for the peak region in one of the concentric fringes

6 $${\langle I\rangle }_{{\rm{A}}{\rm{r}}{\rm{e}}{\rm{a}}}=\frac{1}{2\pi }{\int }_{0}^{2\pi }{I}_{i}({\boldsymbol{S}}(\phi ))\,{\rm{d}}\phi ,\quad |{\boldsymbol{S}}\,(\phi )|={S}_{{\rm{s}}{\rm{p}}{\rm{e}}{\rm{c}}{\rm{k}}{\rm{l}}{\rm{e}}{\rm{a}}{\rm{r}}{\rm{e}}{\rm{a}}},$$

where φ is the polar angle determining the direction of the scattering vector ***S***(φ). The area satisfying the relation │***S***(φ)│ = ***S***_speckle area_ is designated as the ‘speckle area’ in Fig. 2(b).

### Pixel sampling of diffraction intensity

Here we consider the small-angle diffraction from an object with a projection electron density *ρ*(*r*) with the dimensions of *A*_*x*_ × *A*_*y*_. The electron density is defined for *N*_*x*_ × *N*_y_ pixels (Fig. 2(a)). The diffraction intensity *I*(***S***), which is discretized by 2*M* × 2*M* pixels (Fig. 2(b)), is expressed by the Fourier transform of the autocorrelation function of *ρ*(*r*) as

7 $$I({\bf{S}})=K\,\sum _{{n}_{x}=0}^{{N}_{x}-1}\sum _{{n}_{y}=0}^{{N}_{y}-1}[\rho ({\boldsymbol{r}})\otimes {\rho }^{\ast }(-{\boldsymbol{r}})]\,\exp [2\pi i\{({S}_{{\rm{\max }}}\frac{{n}_{Sx}}{M})\cdot ({A}_{x}\frac{{n}_{x}}{{N}_{x}})+({S}_{{\rm{\max }}}\frac{{n}_{Sy}}{M})\cdot ({A}_{y}\frac{{n}_{y}}{{N}_{y}})\}],$$

where *K* is a constant and (*n*_*x*_, *n*_*y*_) is the position of the pixel in the electron density map. Then, the coordinate of the pixel is expressed as ${\boldsymbol{r}}=({A}_{x}\frac{{n}_{x}}{{N}_{x}},\,{A}_{y}\frac{{n}_{y}}{{N}_{y}})$, where (*n*_*Sx*_, *n*_*Sy*_) is the position of the pixel in the reciprocal plane and *n*_*Sx*_ (*n*_*Sy*_) takes values from −*M* to +*M* −1. The scattering vector directing the center of the pixel is written as ${\bf{S}}=({S}_{x},\,{S}_{y})=({S}_{max}\frac{{n}_{Sx}}{M},\,{S}_{max}\frac{{n}_{Sy}}{M})$, where ***S***_max_ is the maximum resolution at the edge of a square-shaped detector. In the experiment, a diffraction pattern recorded by an area detector is divided into a number of pixels. Then, the scattering vector and the diffraction intensities are converted to digitized and integrated quantities, respectively (Fig. 2(c)) (The derivation of Eq. (8) is described in Supplementary Information S2),

8 $$\begin{array}{rcl}{I}_{{\rm{pixel}}}({n}_{Sx},\,{n}_{Sy}) & = & {\int }_{{n}_{Sx}-\frac{1}{2}}^{{n}_{Sx}+\frac{1}{2}}{\int }_{{n}_{Sy}-\frac{1}{2}}^{{n}_{Sy}+\frac{1}{2}}I({\bf{S}}\text{'}){\rm{d}}{n}_{Sx}^{\text{'}}{\rm{d}}{n}_{Sy}^{\text{'}}\quad {\bf{S}}\text{'}=({S}_{{\rm{\max }}}\frac{{n}_{Sx}^{\text{'}}}{M},{S}_{{\rm{\max }}}\frac{{n}_{Sy}^{\text{'}}}{M})\\  & = & K\sum _{{n}_{x}=0}^{{N}_{x}-1}\sum _{{n}_{y}=0}^{{N}_{y}-1}[\rho ({\bf{r}})\otimes {\rho }^{\ast }(-{\bf{r}})]\,{\rm{sinc}}(\pi \frac{{n}_{x}}{M}){\rm{sinc}}(\pi \frac{{n}_{y}}{M})\\  &  & \exp [2\pi i(\frac{{n}_{Sx}}{M}{n}_{x}+\frac{{n}_{Sy}}{M}{n}_{y})]\quad \quad \quad \quad {\rm{sinc}}(x)=\frac{\sin (x)}{x}\end{array}$$

The relation $\frac{{A}_{x}}{{N}_{x}}=\frac{{A}_{y}}{{N}_{y}}=\frac{1}{{S}_{{\rm{\max }}}}$ was used in the derivation. In this study, the sampling of the diffraction intensity by the detector pixels is designated as ‘pixel sampling’. Then, ***I***(***S***) can be calculated from a recorded diffraction pattern^21^ ${I}_{{\rm{p}}{\rm{i}}{\rm{x}}{\rm{e}}{\rm{l}}}({n}_{Sx},\,{n}_{Sy})$ as

9 $$I({\boldsymbol{S}})={\rm{FT}}[\frac{{{\rm{FT}}}^{-1}[{I}_{{\rm{pixel}}}({n}_{Sx},\,{n}_{Sy})]}{{\rm{sinc}}(\pi {n}_{x}/M){\rm{sinc}}(\pi {n}_{y}/M)}]$$

where FT and FT^−1^ represent the discrete Fourier transform and the inverse discrete Fourier transform, respectively. The intensity variation of the speckle peaks is reduced as illustrated in Fig. 2(c). This reduction will affect the speckle contrast value calculated from *I*_pixel_ (*n*_*sx*_, *n*_*sy*_). Although the deconvolution of the two sinc functions in Eq. (9) is necessary to reconstruct ***I***(***S***) from *I*_pixel_ (*n*_*sx*_, *n*_*sy*_), data missing in the small-angle region due to the beamstop (Fig. 1) in a digitized diffraction pattern causes Gibbs fringes^24^ in the discrete Fourier transform.

### Exact sampling of a diffraction pattern missing the small-angle region

To avoid Gibbs fringes, a Gaussian mask with an annular shape, used in the dark-field phase retrieval method^23^, is multiplied by the experimental diffraction pattern (Fig. 2(d)) (see also Supplementary Information S3);

10 $$M({\boldsymbol{S}})=\frac{1}{2\pi }{\int }_{0}^{2\pi }\exp [-\frac{{({\boldsymbol{S}}-{{\boldsymbol{S}}}_{0}(\phi ))}^{2}{\alpha }^{2}}{2}]{\rm{d}}\phi ,\quad |{{\boldsymbol{S}}}_{0}(\phi )|={S}_{{\rm{speckle}}{\rm{area}}},$$

where the area │***S***_0_(φ)│=***S***_speckle area_ is designated as the ‘speckle area’ in Fig. 2(e). Parameter *α* controls the width of the mask. The annular mask is prepared to emphasize one of the concentric rings in the diffraction pattern from gold colloidal particles, and to simultaneously reduce enough the intensity in the small-angle region to approximately zero^23^. Then, the diffraction intensity multiplied by the annular-shaped mask is expressed as

11 $$M({\boldsymbol{S}})\cdot I({\boldsymbol{S}})={\rm{FT}}[\frac{{{\rm{FT}}}^{-1}[M({\boldsymbol{S}})\cdot I{({\boldsymbol{S}})}_{{\rm{pixel}}}]}{{\rm{sinc}}(\pi \,{n}_{x}/M)\,{\rm{sinc}}(\pi \,\,{n}_{y}/M)}]$$

Because *M*(│***S***_0_(φ)│=1) on the peak region of the mask, Eq. (11) is simply reduced to

12 $$I(|{{\boldsymbol{S}}}_{0}(\phi )|)={\rm{FT}}[\frac{{{\rm{FT}}}^{-1}[I{(|{{\boldsymbol{S}}}_{0}(\phi )|)}_{{\rm{pixel}}}]}{{\rm{sinc}}(\pi \,{n}_{x}/M)\,{\rm{sinc}}(\pi \,{n}_{y}/M)}].$$

Therefore, we can calculate *I*(│***S***_0_(φ)│) on ***S***_0_(φ) from pixel-sampled data. Hereafter, this protocol is designated as ‘exact sampling’.

## Results

For SVS measurements, we used monochromatized XFEL pulses focused by a K-B mirror system, the mirrors of which were fabricated to yield a focal spot of a diameter of 2–3 μm^18^. The averaged profile of the XFEL pulse, measured by a knife-edge scan, was approximated as a Gaussian with a full width at half maximum of approximately 2 μm in both the vertical and horizontal directions^14,15,25^ (Supplementary Information S4). When measuring heavily attenuated 200 XFEL pulses (a transmission of 0.02%) by PIN photodiode, the total energy of a single XFEL pulse was estimated to be 10 ± 0.5 μJ, corresponding to 1.1 × 10^10^ X-ray photons with an energy of 5.5 keV.

### Speckle contrast

**Figure 3:**
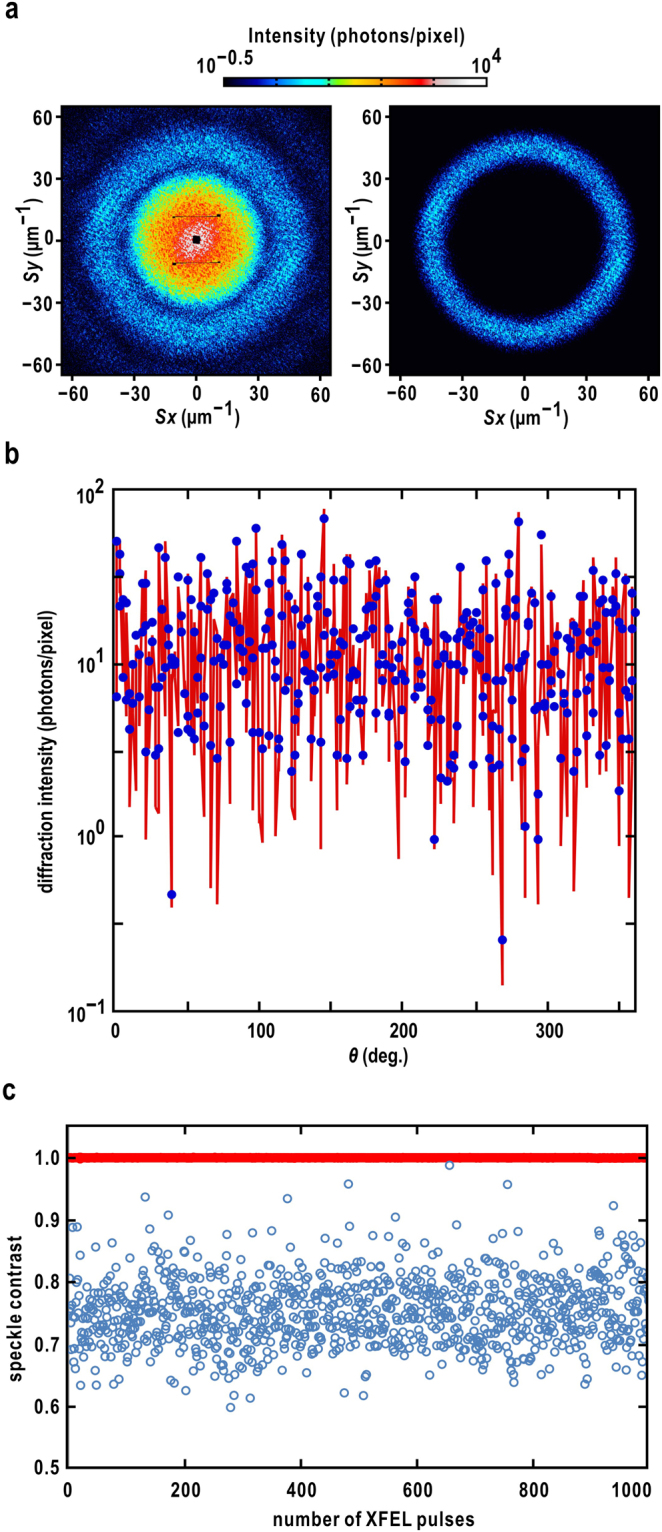
(**a**) Experimental diffraction pattern of gold colloidal particles (left panel), and the masked diffraction pattern (right panel). (**b**) The variation of diffraction intensities along the speckle area on the interference ring in the masked diffraction pattern. The red line is calculated by applying the combination of exact sampling and the annular mask to the pixel-sampled intensity (blue dots). (**c**) Variation of speckle contrast values calculated from 1000 diffraction patterns of gold colloidal particles during the beamtime on March 2015. The diffraction patterns were collected at 30 Hz. Red dots indicate the speckle contrast values calculated by the combination of the exact sampling and the annular mask. Blue circles are the speckle contrast values calculated by the pixel sampling.

Diffraction patterns from gold colloidal particles dispersed uniformly and densely on poly-L-lysine (PLL)-coated silicon nitride membranes were composed of concentric rings accompanying many small speckle peaks (Fig. 3(a)). For the exact sampling, we prepared an annular mask (Eq. (10)) in referring the diffraction patterns and Eq. (4). The peak position of the mask in the radial direction (***S***_speckle area_) was set at 45.2 μm^−1^, where the second interference fringe in the structure factor of a gold colloidal particle had a maximum. In addition, the standard deviation of the mask (*α*) was determined to be 0.11 μm so that only the second interference fringe was enhanced.

Figure 3(b) shows an intensity profile on the speckle area calculated by the proposed protocol. The fluctuation in the profile was larger than that by the pixel sampling, particularly in the valleys between speckle peaks. In the pixel sampling, because the speckle peaks were recorded by a few detector pixels, valleys of real profiles become unclear. Therefore, the deconvolution of the sinc functions from the pixel sampled diffraction patterns by using the exact sampling and the Gaussian mask (Eq. (12)) is necessary to recover the profiles of fine speckle patterns. According to Eq. (3), the larger intensity differences between speckle peaks and valleys give better speckle contrast value.

The estimated speckle contrast of each diffraction pattern was close to 1 during experiments at SACLA from 2014 to 2017 (Table 1). An example of SVS analysis is shown in Fig. 3(c). The speckle contrast values for 1000 XFEL shots were close to one with small fluctuations, with an averaged value of 0.997 ± 0.001. These findings indicated that the focused XFEL pulses were almost complete spatial coherence. In fact, the diffraction patterns of cuboid-shaped cuprous oxide particles (Fig. 1(b)) measured just after the SVS measurement showed visibility consistent with this result (Supplementary Information S1). In contrast, the speckle contrast values given by the pixel sampling displayed large fluctuations from 0.6 to 0.9, with an average of 0.75 ± 0.06. When the K-B mirror was detuned, both calculation methods resulted in speckle contrast values of 0.6–0.8 (Supplementary Information S5).

**Table 1:** Averaged speckle contrast in each beamtime at SACLA.

Beamtime	Averaged speckle contrast^*^
2014 summer	0.996
2014 winter	0.997
2015 winter	0.996
2016 summer	0.996
2016 winter	0.997
2017 summer	0.996

^*^The standard deviation values for the speckle contrast were approximately 0.001 for all measurements.

### Beam size contribution to diffraction pattern

**Figure 4:**
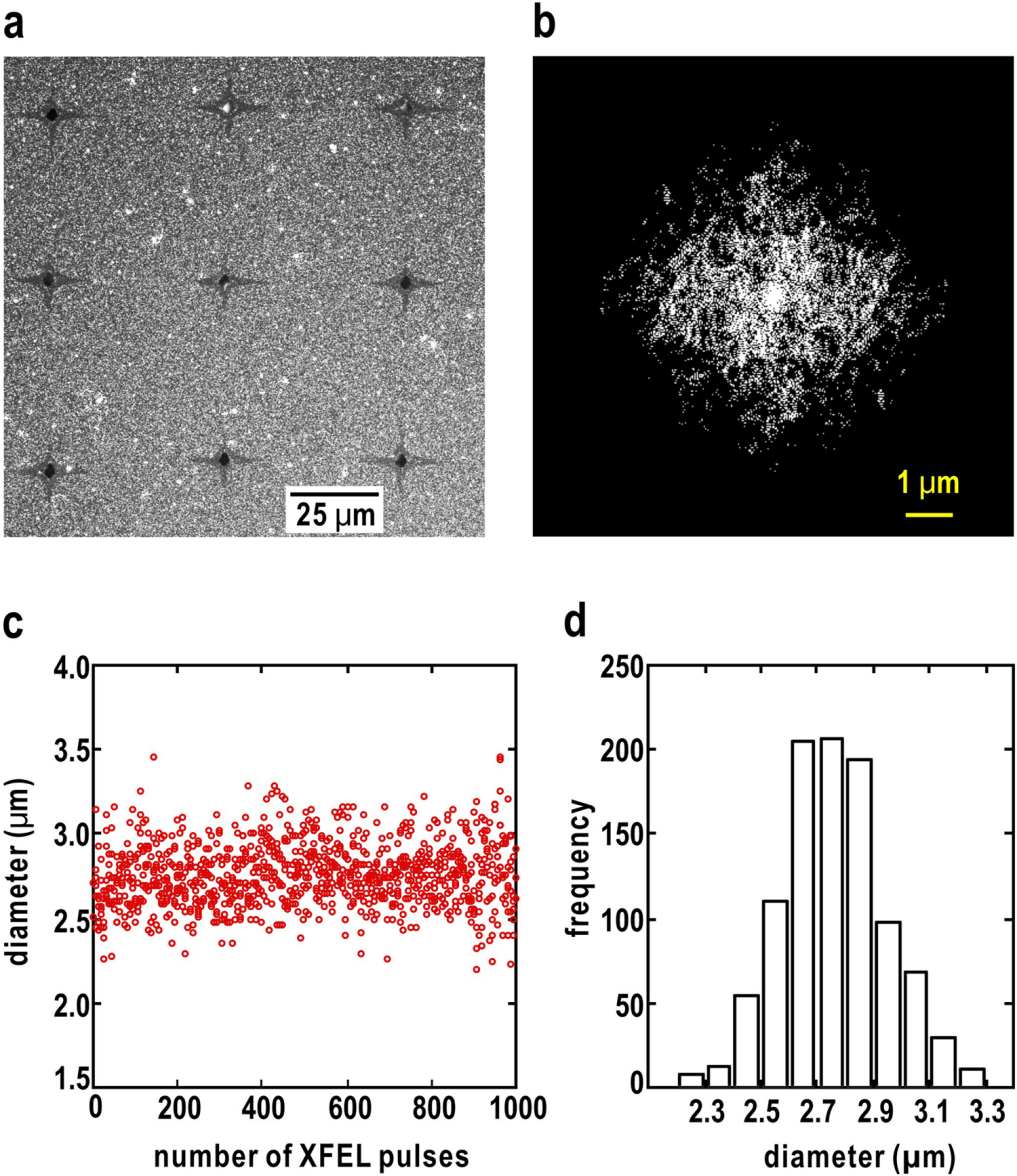
(**a**) A SEM image of a part of the specimen with irradiated spots. (**b**) The binarized autocorrelation function of the diffraction pattern of gold colloidal particles by using Eq. (13). (**c**) Shot-by-shot variation of the approximate sizes of XFEL pulses contributing to the diffraction patterns. (**d**) A frequency distribution of the sizes in panel (c).

After the SVS measurement, the irradiation traces of XFEL pulses on a silicon nitride membrane were viewed with a scanning electron microscope. Each focused XFEL pulse made a single hole with an average size of 6.1 ± 1.9 μm, and removed gold colloidal particles in a cross-shaped area extending 19 μm from the hole (Fig. 4(a)). Because the vaporization of atoms in the focal spots induced cracking of membranes, the size of the hole tended to be larger than that of the vaporization area. In fact, the sizes of the speckle peaks were estimated to be approximately 0.44 μm^−1^, and corresponded roughly to the full-width at half maximum of the beam profile measured by the knife-edge scan (Supplementary Information S4). These results indicate that only a central part of the focused X-rays would predominantly contribute to the observed diffraction pattern. Therefore, the speckle contrast estimated from the diffraction pattern reflects the spatial coherence of an area with sufficient intensity to give the diffraction pattern.

Because the gold colloidal particles are uniformly and densely dispersed on PLL-coated silicon nitride membranes, the area of each focused XFEL pulse contributing to the diffraction patterns can be estimated from the maximum dimensions in the auto-correlation function of the diffraction pattern. In the calculation of the auto-correlation function, we multiplied an annular-shaped mask to the diffraction pattern to significantly reduce the aliasing in the Fourier transform caused by the beamstop region as

13 $$\begin{array}{c}\int M({\boldsymbol{S}}){}^{2}I({\boldsymbol{S}})\,\exp (2\pi i{\boldsymbol{u}}\cdot {\boldsymbol{S}})\,{{\rm{d}}}^{{\rm{2}}}S=\int {\rho }_{M}({\boldsymbol{r}})\,{\rho }_{M}({\boldsymbol{r}}+{\boldsymbol{u}})\,{{\rm{d}}}^{{\rm{2}}}r\\ {\rho }_{M}({\boldsymbol{r}})={{\rm{FT}}}^{-1}[M({\boldsymbol{S}})]\otimes \rho ({\boldsymbol{r}})\end{array}$$

The autocorrelation function from a masked diffraction pattern with little aliasing is easy to be binarized in order to find the shape and size of the specimen area by using a focused X-ray beam (Fig. 4(b)). It should be noted that the size from the autocorrelation function become nearly the twice that of the real beam size. Figure 4(c) shows the variation of the diameter of each XFEL pulse contributing to the diffraction pattern, assuming that the area is approximated by a circle. The size distribution was approximated by a Gaussian with an average of 2.8 μm and a standard deviation of 0.2 μm (Fig. 4(d)). When the beam profile is approximated by a Gaussian (Supplementary Information S4), the beam intensity within the area with a diameter of 2.8 μm is higher than 26% of the peak intensity. Therefore, at least within this area, focused XFEL pulses had a high level of coherence, as demonstrated in Fig. 3(c).

### Degradation of specimen by focused XFEL pulses

**Figure 5:**
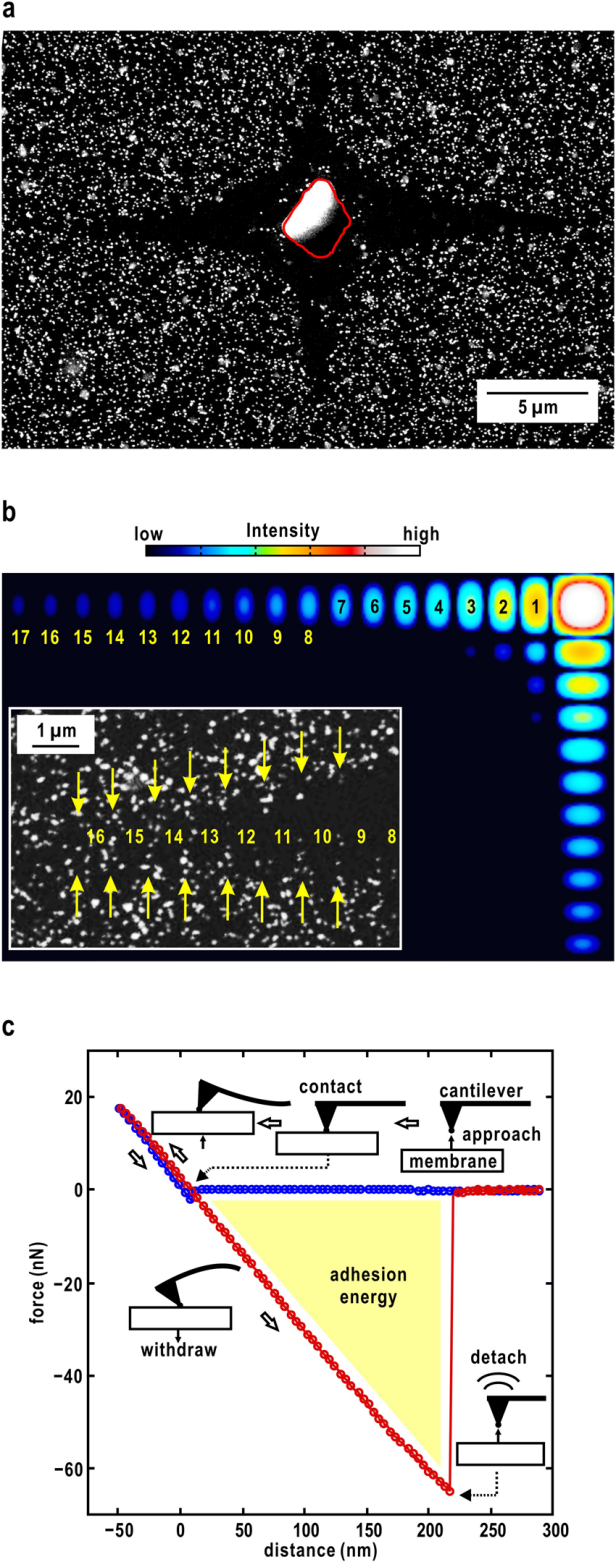
(**a**) A magnified SEM image of an irradiated area. Red line indicates the edge of an area where both gold colloidal particles and the membrane are vaporized. (**b**) A comparison of the theoretical pattern of the focal spot and an SEM image of the tail region of a cross pattern, which shows the repetition between the absence and presence of particles as yellow arrows. The labelled indices are the peak numbers from the center (see Eq. (14)). (**c**) Force-distance curve measured by AFM for the movement of a membrane and the probe of a cantilever which is contacted and removed. Blue and red lines indicate the force-distance curve when the probe is approaching the membrane and when it is withdrawing, respectively. After the probe contacts the membrane, the force applied to the cantilever is gradually increased. When the force reached 18 nN, the probe was removed from the membrane. Because of the adhesion energy between the gold colloidal particle and the membrane, the cantilever was negatively bent until the probe was adsorbed to the membrane. After the removal of the probe from the membrane, the cantilever elastically returned to the initial shape.

The intensity distribution of the focused XFEL pulse at the position (*x*, *y*) in the focal plane is approximated as

14 $${I}_{{\rm{f}}{\rm{o}}{\rm{c}}{\rm{a}}{\rm{l}}{\rm{p}}{\rm{l}}{\rm{a}}{\rm{n}}{\rm{e}}}(x,\,y)={I}_{0}\,{{\rm{s}}{\rm{i}}{\rm{n}}{\rm{c}}}^{2}(\pi \,\frac{a}{\lambda }\frac{x}{{L}_{{\rm{f}}{\rm{o}}{\rm{c}}{\rm{u}}{\rm{s}}}})\,{{\rm{s}}{\rm{i}}{\rm{n}}{\rm{c}}}^{2}(\pi \frac{b}{\lambda }\frac{y}{{L}_{{\rm{f}}{\rm{o}}{\rm{c}}{\rm{u}}{\rm{s}}}}),$$

where *a* and *b* are the acceptance of the K-B mirror in the vertical and horizontal directions, respectively. *I*_0_ is the intensity at the beam center. ***L***_focus_ is the focal length of the K-B mirror. The cross-shaped trace of each XFEL pulse was almost consistent with this theoretical equation (Fig. 5(a)). The central hole with an average dimension of 6.1 ± 1.9 μm was mainly made by the vaporization of both irradiated gold colloidal particles and the silicon nitride membrane at the atomic level. Gold colloidal particles were absent in an area of 3–4 μm width around the edge of the hole. Both the size of the central hole and of the area missing gold colloidal particles depended on the incident intensity, indicating that the threshold intensity would be necessary for vaporization and the dissociation of gold colloidal particles from the membrane.

Along the cross pattern of the focused XFEL pulse, gold colloidal particles were absent at a 580 nm interval up to 13 μm from the beam center. From the acceptance (615 μm and 632 μm in the horizontal and vertical directions, respectively^18^) and the focal length (1.55 m^18^) of the K-B mirror, the repeat distance were calculated to be 551 nm and 567 nm in the vertical and horizontal directions, respectively, which were consistent with those observed in the SEM image shown in Fig. 5(b). Near the outer edge of the region missing gold colloidal particles, a part of the energy provided by a single XFEL pulse is in balance with the adhesion energy of the gold colloidal particles on the membrane. The adhesion energy was estimated by atomic force microscopy (AFM) using a cantilever probe, the tip of which was gold coated and had a diameter of 60 nm, to mimic the adhesion interaction (Fig. 5(b)). From the variation of the force-distance curve during the adhesion and removal of the tip, the adhesion energy of a gold colloidal particle on the membrane was estimated to be 130 fJ (Fig. 5(c)). This result indicates that the energy of a single XFEL pulse near the outer edge of the region missing gold colloidal particles is larger than the measured adhesion energy.

## Discussion

We first discuss the coherence of the XFEL pulses provided at SACLA. The combination of the exact sampling and an annular mask (Eq. (12)) results in a speckle contrast value consistent with the visibility of the diffraction patterns, rather than the pixel sampling (Figs 3 and 4). Detectors with fine pixels or a long camera distance may improve the pixel sampling, but the integrated diffraction intensity becomes small. From the point of view of signal-to-noise ratio, the exact sampling is advantageous for measuring the speckle contrast.

The square root of experimentally measured speckle contrast (Eq. (5)) $\sqrt{C}$ is related with the transverse *β*_*t*_ and the correction factor accounting the effect of the longitudinal coherence *β*_l_ as $\sqrt{C}={\beta }_{t}\,{\beta }_{l}$^20,21^. The correction factor depends on scattering vector and the energy band width of XFEL pulse, and can be calculated for the beam size ***L*** and specimen thickness ***W*** under assuming a Gaussian spectrum to

15 $$\begin{array}{lll}{\beta }_{l}^{2} & = & \frac{2}{{L}^{2}{W}^{2}}{\int }_{0}^{L}{\int }_{0}^{W}(L-x)\,(W-y)\,\{\exp \,[-\pi {(\xi x+\eta y)}^{2}]+\exp \,[-\pi {(\xi x-\eta y)}^{2}]\}\,{\rm{d}}x\,{\rm{d}}y\\ \xi  & = & \frac{{\rm{\Delta }}\lambda }{\lambda }S\sqrt{1-\frac{{S}^{2}{\lambda }^{2}}{4}},\,\quad \quad \eta =\frac{\lambda }{2}\frac{{\rm{\Delta }}\lambda }{\lambda }{S}^{2}\end{array},$$

where Δλ/λ is the bandwidth of the monochromatized XFEL pulse at BL3 of SACLA (5.8 × 10^−5^)^26^. In our experiment, the beam size was estimated to be 2.8 μm, and the specimen thickness was approximately 50 nm. At the speckle area used in this study (*S* = 45.2 μm^−1^, corresponding to diffraction angle 2*θ* = 0.58 deg.), *β*_*l*_ was calculated to be 0.9998, and then *β*_*t*_ was 0.9982 ± 0.0005. The transverse coherence is connected to the number of modes *M*, as ${\beta }_{t}=1/\sqrt{M}$^20,27^ or to the mutual coherence function γ(R = 0, τ = 0) of the field at separation *R* as *β*_*t*_ = │γ(R = 0, τ = 0)│. Therefore, both the estimated mode number and the mutual coherence function were 1.

Although the K-B mirror is tuned prior to the experiments^15^, defocus may occur after several tens of hours from the initial tuning, due, for instance, to a small deformation of the mirror housing. In fact, the misalignment in the K-B configuration regarding the focal length and grazing-incidence angle is known to be one of factors causing defocus^28^. Then, a specimen area irradiated by a large beam size from a misaligned K-B mirror system results in small speckle patterns (Supplementary Information S5). Even using the proposed protocol, the small speckle size would be difficult to recover the profile of speckle patterns and give smaller speckle contrast values with large fluctuations shot-by-shot. However, more experimental evidences are necessary to understand the causes of small speckle contrast values.

To date, two techniques have been proposed to measure the spatial coherence of focused XFEL pulses. One technique evaluates the visibility of the interference diffraction pattern from a pair of gold colloidal particles in a flow of suspension^29^. Because pairs of gold colloidal particles with different sizes are rarely located within an irradiation area of XFEL pulses, the rate at which desired interference patterns are obtained is very small. The other technique also evaluates the visibility of interference diffraction patterns from two pinholes made in the membrane^30^. Because these techniques measure the visibility of accidentally obtained interference patterns, they are difficult to apply to shot-by-shot evaluation of focused XFEL pulses. In addition, the parameter fitting of the weak interference profile from only two scatterers would lead to uncertainty in the estimated visibility. In contrast, in our method, the hit rate of XFEL pulses to specimens is almost 100%, and all diffraction patterns display good signal to noise ratio, because the gold colloidal particles are uniformly and densely dispersed on the membrane.

However, an advantage of these two techniques is the measurement of the position dependent variation of coherence to yield the complex degree of spatial coherence and also global coherence by varying the distance between two scattering objects, while the present method evaluated the their transverse coherence of the monochromatized and focused XFEL pulses. Therefore, improvement in specimen preparation for controlling the area of each focused XFEL pulses by, for instance, changing the diameter of the gold colloidal particles, would allow us to measure coherence as a function of the area size.

Next, we discuss the radiation damage of specimens by the tail region of the focused XFEL pulses. As demonstrated in Fig. 5(a), the trace is consistent with the theoretically expected profile (Eq. (14)). From a PIN-photodiode measurement providing the integrated value of Eq. (14), ${I}_{0}\frac{\lambda }{a}{L}_{{\rm{focus}}}\frac{\lambda }{b}{L}_{{\rm{focus}}}$, the energy of *I*_0_ is estimated to be approximately 32 μJ/μm^2^, corresponding to 3.6 × 10^10^ X-ray photons with an energy of 5.5 keV per 1 μm^2^. In the area contributing to the diffraction pattern (approximately 2.5 μm in diameter), the integrated pulse energy is approximately 7.4 μJ. Outside the area, the tail region of the focused XFEL pulse still has the power to degrade the specimen as the removal of gold colloidal particles with an adhesion energy of 130 fJ to the membrane.

Radiation damage of the specimens by laser pulses with a wavelength in the visible region are intensively studied, and various mechanisms inducing the degradation of specimens are proposed, such as Coulombic explosion^31^, bubble nucleation^32^, rapid thermal annealing^33,34^, megasonic vibration^35^, and the thermoelastic effect^32^. In the former two, plasma and/or electrons appear in specimens irradiated by laser pulses. In the X-ray region, the photoelectric effect would be a major cause of radiation damage. According to the literature on the photoelectric effect^36^, in our SVS measurement, 5% of X-ray photons at 5.5 keV absorbed by a 50 nm gold colloidal particle are used for the photoelectric effect. In a 100-nm-thick silicon nitride membrane, 0.4% of the X-ray energy goes towards the photoelectric effect. Then, as a mechanism for the removal of gold colloidal particles from the membrane (Fig. 5), the electrostatic repulsion between both gold colloidal particles and the membrane charged by the photoelectric process would pull gold colloidal particles away.

To irradiate all fresh specimens with XFEL pulses, the tail region of the focused XFEL pulses must be taken into consideration. In the irradiation of specimens by XFEL pulses, for instance, the step width of the raster scan or flow speed of the specimen solution in a liquid jet are important factors to consider in avoiding the degradation of the specimens outside the peak area. When the speed is slow, specimen particles are irradiated by the tail region of the focused XFEL pulses. When specimens are injected into the irradiation area from a vertical or horizontal direction, specimens must flow at a speed faster than the width of the tail region per pulse. For instance, in our experiments, to avoid degradation by the tail region, the raster scan step is set to be 25–50 μm for every pulse provided at a rate of 30 Hz^25^. In the next generation XFEL facility, where a 1 MHz repetition rate will be achieved by using superconducting magnets, each pulse provided with an incident intensity used in this study requires more rapid movement of the specimens to collect data from intact specimens. As a possible method to reduce or avoid the degradation of specimens, the injection of specimen particles at an inclined geometry would be more effective than those from the vertical or horizontal direction when using KB-type focusing mirror optics.

## Methods

### Specimen preparation

Specimens were prepared by dispersing gold colloidal particles with diameters of 50 nm (British Biocell International Solutions, UK) on a silicon nitride membrane with a thickness of 100 nm^17^. To increase the affinity of the particles to the membrane, the membrane was coated with PLL after the deposition of a thin carbon layer with an approximate thickness of 30 nm. To uniformly disperse gold colloidal particles, we used an electrospray device (PDS-D01, Hamamatsu Nano Technology, Japan). A suspension of gold colloidal particles is applied into a thin capillary tube with an exit nozzle of an inner diameter of 24 μm. The voltage between the electrode inside the capillary and the membrane was adjusted to 5000 V. It took approximately 30 min until the number density reached approximately 50 particles/2 × 2 μm^2^ on the membrane. After the preparation, the number density was checked by using a scanning electron microscope (SEM) (TM3000, Hitachi High-Technologies, Japan).

### Diffraction data collection

We conducted CXDI experiments with the prepared specimens using focused X-ray pulses at BL3 of SACLA^37^. X-ray pulses provided with photon energies of 5.47 ± 0.01 keV were monochromatized and focused with a K-B focusing mirror system^18^. Diffraction patterns were collected by using our custom-made diffraction apparatus KOTOBUKI-1^14^ or TAKASAGO-6^25^ (Fig. 1(b)). The diffraction apparatus was placed so that the specimen position was within the focal spot, which was confirmed by the Foucault knife-edge test with a thin gold wire. The size of the spot was in the range of 1.9–2.1 μm, determined by the full width at half maximum as measured by the knife-edge scan method. Background scattering from upstream optics was eliminated by a pair of slits composed of L-shaped silicon frames with beveled edges^14,15,25^.

Because focused single X-ray pulses destroy particles at the atomic level, fresh gold colloidal particles are supplied into the illumination area of the focused X-ray pulse trains by scanning each silicon nitride membrane at a step of 25–50 μm/pulse in the horizontal and vertical directions. In the preliminary stage of this study, diffraction patterns were collected at 1 Hz by using the KOTOBUKI-1 apparatus equipped with a high-precision stage^14^. Later, the TAKASAGO-6 apparatus equipped with a high-speed translation stage allowed us to collect diffraction patterns at 30 Hz^25^. Each scanning motion was started with the signal provided from an accelerator control system for triggering XFEL pulses.

Diffraction patterns were recorded by two multi-port CCD (MPCCD) detectors^38^ (Fig. 1(b)). A MPCCD-Octal detector composed of 8 CCD panels is placed approximately 1.6 m downstream from the specimen position to record diffraction patterns in the resolution range of 7–210 nm. The central aperture of the MPCCD-Octal detector was set at 8.0 mm. A MPCCD-Dual detector composed of two CCD panels was placed at 3.2 m downstream from the specimen position to record patterns in the resolution range of 80–500 nm. Direct XFEL pulses were blocked by a beam stop of 2 × 2 mm^2^. Small-angle diffraction patterns were collected by attenuating the diffraction intensity using an aluminium foil of 15–100-μm thick placed in front of the MPCCD-Dual detector.

### Data processing

Diffraction patterns collected by the two MPCCD detectors were processed automatically after the finish of each scan by the *G-SITENNO* program suite^39,40^ on a supercomputer composed of 960 Intel Xeon(R) CPU X5690 (3.47 GHz/core) cores at SACLA^41^. In every beamtime, we determined the camera parameters by using a diffraction pattern from a single cuboid-shaped cuprous oxide particle with a dimension of approximately 500 nm^39^. After subtracting the dark currents of the detectors, the diffraction patterns recorded by the two detectors were merged into a single file. A program for determining the visibility using Eqs (9), (10) and (12) was originally coded by using the FORTRAN language. The program can process 1000 diffraction patterns in 1 min.

### Atomic force microscopy

The adhesion energy of a single gold colloidal particle to the surface of the PLL-coated silicon nitride membrane was measured by using a SP400 atomic force microscope (Seiko Instruments, Japan) with a cantilever, which had a gold-coated probe with a diameter of 60 nm (BL-RC150VB-C1, Olympus, Japan). The adhesion energy of the gold colloidal particle to the membrane was estimated by measuring the force-distance curve between the probe and the membrane according to a literature^42^.

## Electronic supplementary material

Supplementary Information
