# Supplementary material for: Shot-by-shot characterization of focused X-ray free electron laser pulses
Source: Sci Rep. 2018 Jan 16;8:831. doi: 10.1038/s41598-018-19179-3 (PMC5770435; doi:10.1038/s41598-018-19179-3)
Supplement: Supplementary file 1 — Supplementary Information [file 41598_2018_19179_MOESM1_ESM.pdf]

Supplementary Information for

“Shot-by-shot characterization of focused X-ray free electron laser pulses”

Amane Kobayashi[1,2], Yuki Sekiguchi[1,2], Tomotaka Oroguchi[1,2], Masaki Yamamoto[2], and Masayoshi Nakasako[1,2]*

[1] Department of Physics, Faculty of Science and Technology, Keio University, 3-14-1 Hiyoshi, Kohoku-ku, Yokohama, Kanagawa 223-8522, Japan

[2] RIKEN SPring-8 Center, 1-1-1 Kouto, Sayo, Hyogo 679-5148, Japan

Corresponding author

E-mail: nakasako@phys.keio.ac.jp; Fax, +81-45-5666-1672.

Supplementary Information S1

The visibility of a diffraction pattern from a single cuboid-shaped cuprous oxide particle was measured (Fig. S1). The line profiles along the cross-shaped flares are shown in Fig. S1. At the valleys in the profile, the diffraction intensities were close to zero. Therefore, the visibility of this diffraction pattern was almost 1, and then the spatial coherence of the focused XFEL pulse was almost complete. In this diffraction pattern, because the profile of each speckle peaks were recorded by more than ten detector pixels, the diffraction intensities at valleys between speckle peaks were correctly evaluated. In contrast, in the speckle visibility spectroscopy measurements for a number of small gold colloidal particles, the speckle peaks are as small as a few detector pixels. Therefore, diffraction intensities at peaks and valleys in the speckle patterns are difficult to be correctly measured by the pixel sampling method.

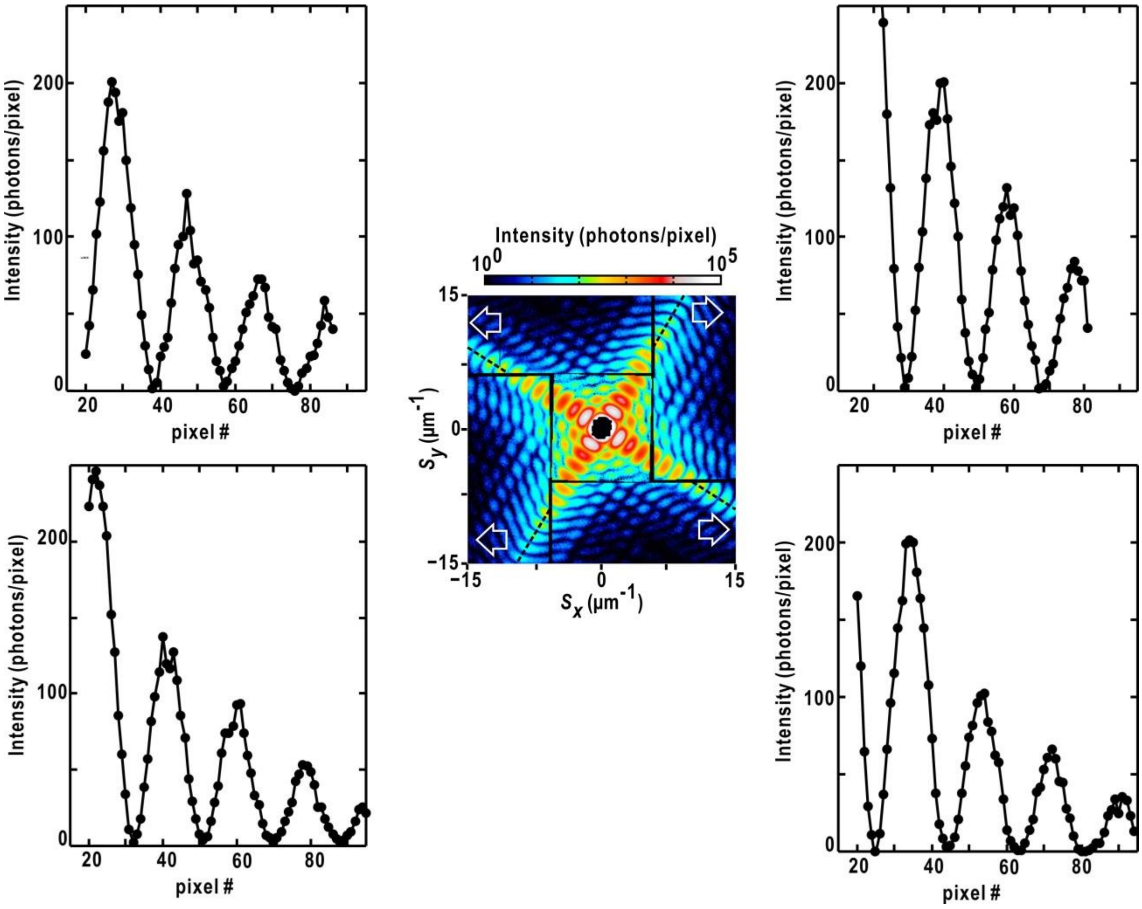

Figure S1

Line profiles along the flares in the diffraction pattern from a cuprous oxide particle. The profiles were obtained along the four flares.

Supplementary Information S2

Eq. (8) is derived as follows.

$$I_{\text{pixel}}(n_{Sx}, n_{Sy}) = \int_{n_{Sx}-\frac{1}{2}}^{n_{Sx}+\frac{1}{2}} \int_{n_{Sy}-\frac{1}{2}}^{n_{Sy}+\frac{1}{2}} I(\boldsymbol{S}') \mathrm{d}n'_{Sx} \mathrm{d}n'_{Sy} \qquad \boldsymbol{S}' = \left(S_{max} \frac{n'_{Sx}}{M}, S_{max} \frac{n'_{Sy}}{M} \right)$$

$$= \int_{n_{Sx}-\frac{1}{2}}^{n_{Sx}+\frac{1}{2}} \int_{n_{Sy}-\frac{1}{2}}^{n_{Sy}+\frac{1}{2}} K \sum_{n_x=0}^{N_x-1} \sum_{n_y=0}^{N_y-1} [\rho(\boldsymbol{r}) \otimes \rho^*(-\boldsymbol{r})] \times$$

$$\exp\left[2\pi i \left\{ \left(S_{\max} \frac{n'_{Sx}}{M} \right) \cdot \left(A_x \frac{n_x}{N_x} \right) + \left(S_{\max} \frac{n'_{Sy}}{M} \right) \cdot \left(A_y \frac{n_y}{N_y} \right) \right\} \right] \mathrm{d}n'_{Sx} \mathrm{d}n'_{Sy}$$

$$= K \sum_{n_x=0}^{N_x-1} \sum_{n_y=0}^{N_y-1} [\rho(\boldsymbol{r}) \otimes \rho^*(-\boldsymbol{r})] \int_{n_{Sx}-\frac{1}{2}}^{n_{Sx}+\frac{1}{2}} \exp\left[2\pi i \left\{ \left(S_{\max} \frac{n'_{Sx}}{M} \right) \cdot \left(A_x \frac{n_x}{N_x} \right) \right\} \right] \mathrm{d}n'_{Sx}$$

$$\int_{n_{Sy}-\frac{1}{2}}^{n_{Sy}+\frac{1}{2}} \exp\left[2\pi i \left\{ \left(S_{\max} \frac{n'_{Sy}}{M} \right) \cdot \left(A_y \frac{n_y}{N_y} \right) \right\} \right] \mathrm{d}n'_{Sy}$$

The integration regarding n_{Sx}' becomes

$$\int_{n_{Sx}-\frac{1}{2}}^{n_{Sx}+\frac{1}{2}} \exp\left[2\pi i \left\{ \left(S_{\max} \frac{n'_{Sx}}{M} \right) \cdot \left(A_x \frac{n_x}{N_x} \right) \right\} \right] \mathrm{d}n'_{Sx}$$

$$= \int_{n_{Sx}-\frac{1}{2}}^{n_{Sx}+\frac{1}{2}} \exp[2\pi i\, G\, n'_{Sx}] \mathrm{d}n'_{Sx} \qquad G = \left(\frac{S_{\max}}{M} \right) \cdot \left(A_x \frac{n_x}{N_x} \right)$$

$$= \frac{1}{2\pi i G} \left[\exp\left(2\pi i\, G\, n'_{Sx} \right) \right]_{n'_{Sx}=n_{Sx}-\frac{1}{2}}^{n'_{Sx}=n_{Sx}+\frac{1}{2}} = \frac{1}{2\pi i G} 2i \sin(\pi G) \cdot \exp(2\pi i G n_{Sx})$$

$$= \mathrm{sinc}\left[\pi \left(\frac{S_{\max}}{M} \right) \cdot \left(A_x \frac{n_x}{N_x} \right) \right] \cdot \exp\left[2\pi i \left\{ \left(S_{\max} \frac{n_{Sx}}{M} \right) \cdot \left(A_x \frac{n_x}{N_x} \right) \right\} \right]$$

$$\mathrm{sinc}(x) = \frac{\sin(x)}{x}, \quad \frac{A_x}{N_x} = \frac{A_y}{N_y} = \frac{1}{S_{\max}}$$

$$= \mathrm{sinc}\left(\pi \frac{n_x}{M} \right) \cdot \exp\left[2\pi i \left(\frac{n_{Sx}}{M} n_x \right) \right]$$

In the course of the calculation, we used the relation on the maximum resolution in the reciprocal space, dimension of projection electron density and the number of pixels to describe the density map. By substituting the integration result into the equation on the diffraction intensity, we obtained the following relation.

$$I_{\text{pixel}}(n_{Sx}, n_{Sy}) = K \sum_{n_x=0}^{N_x-1} \sum_{n_y=0}^{N_y-1} [\rho(\boldsymbol{r}) \otimes \rho^*(-\boldsymbol{r})]$$

$$\mathrm{sinc}\left(\pi \frac{n_x}{M} \right) \mathrm{sinc}\left(\pi \frac{n_y}{M} \right) \exp\left[2\pi i \left(\frac{n_{Sx}}{M} n_x + \frac{n_{Sy}}{M} n_y \right) \right]$$

Supplementary Information S3

Equation (10) can be analytically expressed by using the modified Bessel function of the first kind $\kappa_{\nu}(z)$ as

$$M(\boldsymbol{S})=\int_0^{2\pi}\exp\left(-\frac{(\boldsymbol{S}-\boldsymbol{S}_0(\varphi))^2\alpha^2}{2}\right)\mathrm{d}\varphi, \quad |\boldsymbol{S}_0(\varphi)|=S_{\mathrm{specklearea}}$$

$$=\exp\left(-\frac{\left(|\boldsymbol{S}|^2-|\boldsymbol{S}_0(\varphi)|^2\right)\alpha^2}{2}\right)2\int_0^{\pi}\exp\left(\alpha^2\boldsymbol{S}\cdot\boldsymbol{S}_0(\varphi)\right)\mathrm{d}\varphi$$

$$=\exp\left(-\frac{\left(|\boldsymbol{S}|^2-S_{\mathrm{specklearea}}{}^2\right)\alpha^2}{2}\right)2\pi\,\kappa_0\left(\alpha^2|\boldsymbol{S}|\,S_{\mathrm{specklearea}}\right)$$

$$\left(\kappa_{\nu}(z)=\frac{1}{\pi}\int_0^{\pi}e^{z\cos\theta}\cos\nu\theta\,\mathrm{d}\theta-\frac{\sin\nu\pi}{\pi}\int_0^{\infty}e^{-z\cosh(t)-\nu t}\,\mathrm{d}t\right)$$

Supplementary Information S4

The intensity of the focused XFEL pulse is theoretically described by Eq. (14) in the main text. The central peak of the theoretical intensity profile can be approximated by a Gaussian function as shown in Fig. S2(a). In addition, the experimentally obtained averaged intensity profiles for 200 XFEL pulses with an attenuation of 0.02% were also approximated with Gaussian function both in the horizontal (Fig. S2(b)) and vertical (Fig. S2(c)) directions. Therefore, we approximated the beam profile of the focused XFEL pulses by Gaussian functions in this study.

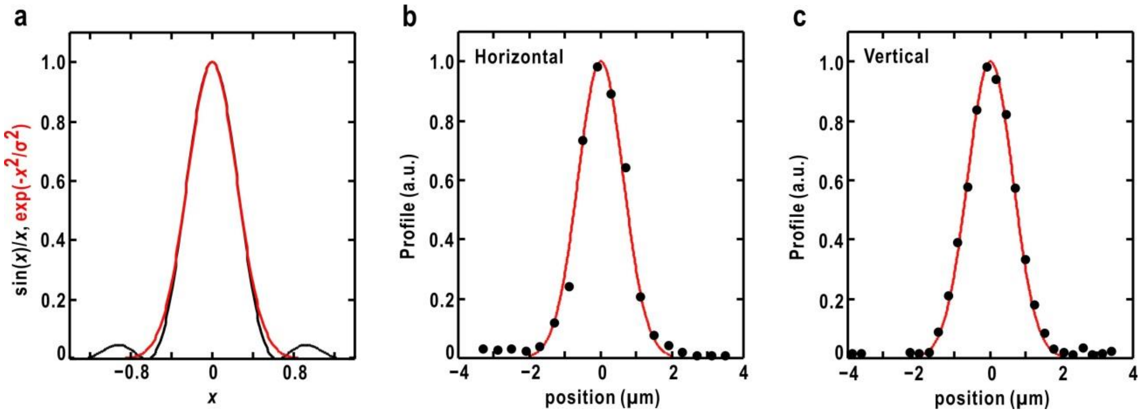

Figure S2

(a) A comparison of the profiles of the main peak in the theoretical profile of focused XFEL pulse (black line) and a Gaussian function (red line). The total intensity and the full width at half maximum of the main peak are well explained by the approximation. For profiles of 200 attenuated XFEL pulses measured by the knife-edge scan method, Gaussian functions (red lines) approximated well the beam profiles (filled circles) both in the horizontal (b) and vertical (c) directions.

Supplementary Information S5

The focal length and grazing-incidence angle of the K-B mirror system must be tuned with the accuracies of micrometers and microradians. When using a misaligned K-B mirror system, the speckle contrast values were significantly lower than those for a well-tuned K-B mirror system (Fig. S3(a)). The misaligned K-B mirror gave XFEL pulses having probably two peaks at the focal region as the traces observed by a scanning electron microscope (Fig. S3(b)). Interestingly, the speckle contrast values from the pixel sampling were close to those reported by Lehmkühler *et al*[21]., who conducted the SVS measurements at SACLA in 2013 near our experiments.

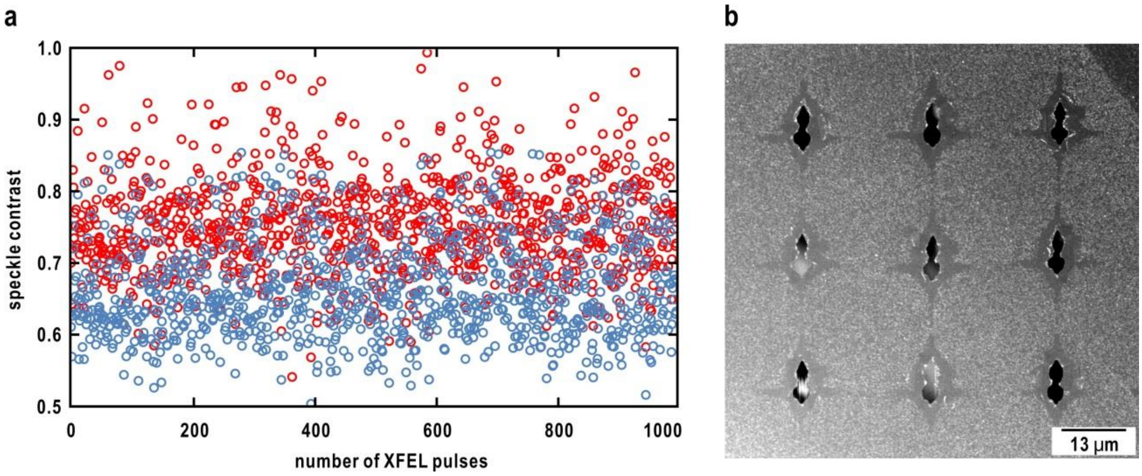

Figure S3

(a) The variation of the speckle contrast values of diffraction patterns taken on July 2013. In the beamtime, the tuning of the K-B mirror was under the influence of temperature fluctuation (±5 K) inside the experimental hutch. Then, the XFEL pulses displayed split at the focal spot as confirmed by SEM observation (b). The speckle contrast from the exact sampling displayed a large fluctuation as well as those from the pixel sampling. The averaged value from the exact sampling was 0.76±0.08 and slightly higher than that of the pixel sampling (0.66±0.07).
